# The Delphi of ORACLE: An Expert Consensus Survey for the Development of the Observational Risk Assessment of Contractures (Longitudinal Evaluation)

**DOI:** 10.1177/02692155241229285

**Published:** 2024-02-08

**Authors:** Hina Tariq, Kathryn Collins, Joel Dunn, Desiree Tait, Sam Porter

**Affiliations:** 1 170790 Faculty of Health and Social Sciences, 6657 Bournemouth University, Bournemouth, UK; 2 Community Therapy Team (Christchurch, Bournemouth & Poole), 1884 Dorset Healthcare University Foundation Trust, Poole, UK

**Keywords:** Contractures, risk assessment, joint mobility, care homes, adults

## Abstract

**Objective:**

Despite rising prevalence rates, no standard tool is available to identify individuals at risk of developing contractures. This study aimed to gain expert consensus on items for the development of the Observational Risk Assessment Tool for Contractures: Longitudinal Evaluation (ORACLE) for care home residents.

**Design:**

A two-round, online modified Delphi study.

**Participants:**

Panellists were qualified healthcare professionals with a background in physiotherapy, occupational therapy, nursing, and rehabilitation medicine.

**Main outcome measures:**

In the first round, the experts were asked to rate the predesigned list of items on a Likert scale while in the second round, consensus was sought in the areas of disagreement identified in the previous round.

**Results:**

The two rounds of the Delphi survey included 30 and 25 panellists, respectively. The average clinical and academic experience of the panellists was 22.2 years and 10.5 years, respectively. The panel demonstrated a high level of consensus regarding the clinical factors (10 out of 15 items); preventive care approaches (9 out of 10 items), and contextual factors (12 out of 13 items) ranging from 70% to 100%.

**Conclusion:**

This Delphi study determined expert consensus on items to be included in a contracture risk assessment tool (ORACLE). The items were related to factors associated with joint contractures, appropriate preventive care interventions, and potentially relevant contextual factors associated with care home settings. The promise of a risk assessment tool that includes these items has the capacity to reduce the risk of contracture development or progression and to trigger timely and appropriate referrals to help prevent further loss of function and independence.

## Introduction

Contractures, commonly defined as restrictions in the passive joint range, are preventable but debilitating consequence of prolonged immobility, eventually leading to structural abnormalities within the impacted joint.^1^ This can lead to further deterioration in the limb and joint flexibility and physical mobility, potentially leading to further physical impairments, decreased independence with everyday activities, and reduced quality of life.^2,3^ Contractures may vary from marginal restriction at a single joint to severe limitations in the range of motion affecting several joints simultaneously.^4^ Based on the severity of functional loss at a joint, contractures can be categorised as (i) severe, (ii) moderate or clinically relevant, or (iii) clinically non-relevant.^5^ The development of progressive joint contractures often follows an insidious pattern, and their initial progression is neither painful nor disabling. Joints only become painful when stretched beyond the point of soft-tissue restriction. For this reason, contractures are often unrecognised by individuals and their caregivers until they become clinically relevant, that is, start interfering with daily functional activities.^6^

Evidence suggests that individuals living in long-term care facilities are predominantly sedentary^7^; hence, they are at a higher risk of developing contractures.^8^ Long-term care settings demonstrate a considerable variation in the prevalence of contractures spanning from 22% to 75% in at least one joint.^4,9–12^ Contracture prevalence was found to be higher in the upper extremities compared with the lower extremities,^4,10^ with the shoulder and knees being the most commonly affected joints.^4^ When considering the impact of mobility on contracture development, there is evidence that 70.5% of non-ambulatory care home residents developed a contracture compared with the ambulatory group, which developed none.^13^

Structured risk assessments play an important role in referring patients to the appropriate healthcare practitioner and enacting early treatment strategies to reduce the risk of the condition progressing. In addition, standard risk assessments are also vital to offer appropriate guidance for risk protection and to have confidence in the tool being used.^14^ The need for a structured and systematic risk assessment of individuals at risk of developing contractures has been identified in the literature.^15^ Despite the reported high prevalence rates, there is a clear lack of a standard, evidence-based measure that can actively identify individuals at risk of developing contractures or worsening of existing contractures in long-term facilities and trigger appropriate and timely referrals to healthcare professionals.

The aim of the current study was to systematically establish the components of Observational Risk Assessment for Contractures: Longitudinal Evaluation (ORACLE) for care home residents, based on multidisciplinary healthcare expert consultation and consensus. The aimed users of the tool will be a range of staff, including healthcare assistants and registered nurses, who are the primary care providers in a care home. During the delivery of care, their regular clinical observations are vital in order to identify the individuals at risk of developing contractures. ORACLE will potentially translate the clinical observations of the care home staff in a systematic fashion, thereby ensuring consistency in identifying the risk, calibrating that risk, helping them prescribe a set of actions in response to the level of risk, and tracking subsequent changes in the risk regularly.

## Methods

This study presents the second stage of a research project which employs the three-stage method for the development and validation of a scale.^16,17^

The purpose of the proposed tool is to support the care home staff in assessing the risk of joint contractures to residents through the application of algorithms to professionally appropriate clinical observations and to respond appropriately to their assessments. Rather than involving a one-off assessment, it is proposed that the tool will be applied repeatedly over time as part of standard clinical observations. In order to tailor it to the care home context where thorough medical assessments might not always be feasible, it has been developed with an emphasis on observable and physically examinable factors rather than on the identification of medical conditions and comorbidities that could contribute to the development of contractures.

A prototype of the tool was originally drafted by a cross-organisational and multi-disciplinary working group led by Dorset Healthcare including physiotherapists, occupational therapists, and registered nurses.

### Study team

The study team comprised the authors of this study, who were responsible for developing and reviewing the proposed questionnaire items and making collective decisions related to the methodology and data analysis. The team consisted of a PhD student, a physiotherapy academic, two nursing academics, and a clinical physiotherapist.

### Study design

This study used the Delphi technique to achieve consensus on the components of ORACLE for care home residents. The Delphi method^18^ is an iterative approach seeking expert opinions and collective agreement from a panel of experts on complex health problems through a series of structured questionnaires.^19^ This study conforms to guidelines for Conducting and Reporting of Delphi Studies.^20^

For this study, a modified Delphi method of two rounds was employed to achieve consensus on different elements of ORACLE. In contrast to the classical Delphi technique, which utilises an initial idea generation phase with open-ended questions, this study employed a pre-designed list of items for the first round.

This list was developed on the basis of the findings of a consensus-based clinical workgroup which developed a prototype tool, a previously conducted systematic review of studies identifying factors associated with joint contractures in adults^21^; standards of proficiency for nursing care^22^; a scoping review of previous reviews of contracture management and prevention^15,23–25^;and the agreement of study team members. This is a commonly accepted modification for the first round of Delphi with a pre-determined list of items based on research evidence or previous knowledge.^18,26,27^

### Participants

There are no guidelines on optimum sample sizes for the Delphi survey; however, previous research has indicated that 64% of Delphi studies included between 11 and 50 participants.^28^ Given the specific focus of this research, this study aimed to recruit up to 30 international panellists representing different geographical and cultural settings to ensure gathering a broad range of opinions.

Expert panellists were invited based on purposive sampling and their expertise in research and clinical practice related to joint contractures. Their eligibility was pre-defined in line with the recommendations for Conducting and Reporting of Delphi Studies (CREDES).^20^

The study invited qualified healthcare professionals with backgrounds in physiotherapy, occupational therapy, nursing, and rehabilitation medicine if they fulfilled one of the following eligibility criteria:

1. At least five years of clinical experience in providing frequent (once every six months) care to adults with joint contractures or
2. Published at least one peer-reviewed research paper on joint contractures.

Healthcare professionals involved in the development of the prototype tool and study team members were excluded. Where contact details could be obtained, authors of research papers identified in stage one (systematic review) were invited to participate. Additionally, the study team members sent email invitations to practising clinicians with recognised clinical expertise in joint contractures. The invitation email contained a summary of the proposed research and the significance of participating in both rounds to reduce attrition bias.^29^ A reminder email was sent if there was no response to the first invitation after three weeks. If there was no response after the second invitation, it was assumed that the participant was unavailable, and no further attempts were made to contact the participant.

### Definition of consensus

Consensus was defined a priori as a percentage agreement threshold ≥70% for both Likert and binary scale responses. This agreement threshold is consistent with other Delphi studies.^28^ The Likert scale scores ranging from 5 to 7 and 1 to 3 were grouped as important/relevant and unimportant/irrelevant, respectively. For example, if ≥70% of the panellists rated an item between 5 and 7, a consensus was reached that the item under consideration was important for the tool. Items that achieved consensus in the first round were excluded in the second round and if agreement was not achieved after two rounds, the items were excluded from the tool.

### Ethics and consent

Ethics approval was obtained from the Research Ethics Committee at Bournemouth University (Ethics identification number: 36403). Participants were given a participant information sheet and provided written informed consent prior to the first round of the survey.

### Data collection procedure

**Figure 1.**
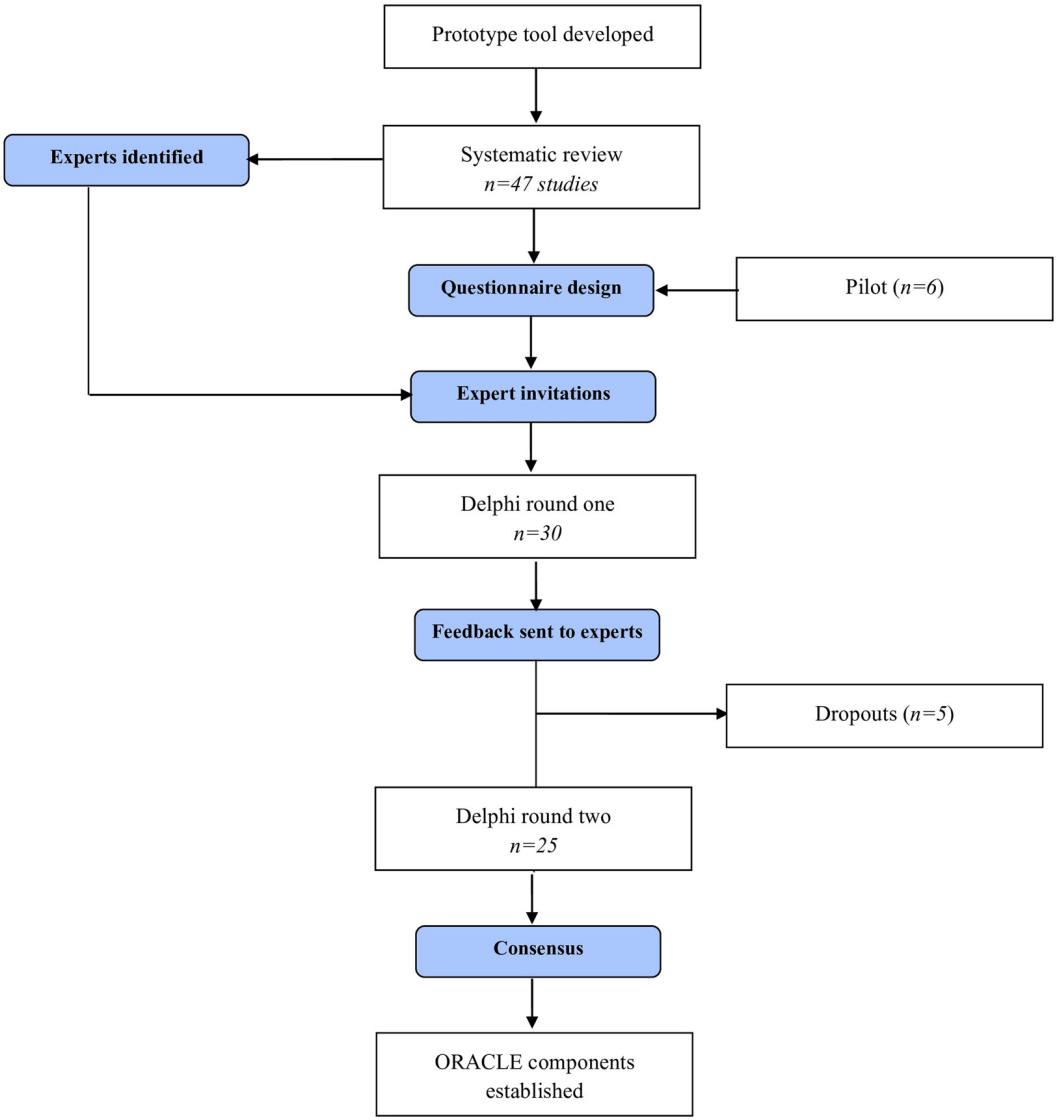
Delphi flow diagram.

A step-by-step process of the study and an overview of the Delphi rounds can be found in Figure 1 and Table 1, respectively.

**Table 1.** Overview of the Delphi rounds.

Round 1: Consultation round	Panellists rated the pre-determined list of items
	Panellists suggested additional items
Round 2: Consensus round	Panellists received structured feedback on a previous round
	Panellists reviewed their ratings in the context of the given feedback and overall group results to reach a consensus
	Panellists rated the additional items identified in the previous round

The draft of the survey questionnaire and the participant information sheet was piloted with six physiotherapists before the commencement of the first round. The aim was to obtain input on content, survey design, clarity of instructions, language, ease of completing the survey, estimated time taken, and other general comments. The feedback received was collated, minor revisions were made accordingly, and the first round of the survey was launched. 

JISC (https://www.jisc.ac.uk/online-surveys), an encrypted online survey platform, was used to construct and distribute both rounds of the Delphi survey.

The first iteration of the Delphi survey was conducted from May 2021 to July 2021. The questionnaire was structured into three sections: (1) development, progression, and identification of joint contractures, (2) preventive care approaches, and (3) panel demographics. After each question, the panellists had the opportunity to suggest additional items and add comments.

1. *Development, progression, and identification of joint contractures:* This section was further categorised into four sub-sections: (A) development of joint contractures, (B) progression of joint contractures, (C) identification of joint contractures, and (D) contextual factors. In subsections A and B, the panellists were provided with a pre-determined list of factors associated with the development and progression of joint contractures separately based on the findings of a previously conducted systematic review^21^ and were asked to rate their importance. A 7-point Likert scale was used: from 1 (extremely unimportant) to 7 (extremely important). These factors build the first part of ORACLE, identifying individuals at risk of developing or worsening contractures. In subsection C, the panellists were asked if different healthcare professionals (physiotherapists, occupational therapists, nurses, and healthcare assistants), families, and informal carers have the ability to identify the clinical factors listed in sections a and b during informal clinical observations. In subsection D, the panellists were provided with a pre-determined list of contextual factors based on the literature^15,23,24^ that may be relevant in the development or progression of joint contractures in a care home setting. Contextual factors are characteristics of the environment or unique factors that have the potential to influence health outcomes.^30^ Panellists were asked to rate the relevance of the contextual factors on a 7-point Likert scale from 1 (extremely irrelevant) to 7 (extremely relevant). Identification of relevant contextual factors will improve the usability and practical implementation of ORACLE in a care home setting.
2. *Preventive care approaches:* In this section, the panellists were asked to rate different evidence-based preventive care approaches^22,25^ important in preventing the development or progression of joint contractures in a care home setting. A 7-point Likert scale was used: from 1 (extremely unimportant) to 7 (extremely important). These care approaches build the second part of ORACLE which provides guidance for the care home staff to intervene in response to the level of risk identified in the first part.
3. *Participant demographics:* Panellists in the last section indicated their country of origin, professional background, highest completed qualifications, practice setting, and years of clinical and academic experience.

After the first iteration was completed, the data was analysed as described below. The second iteration was piloted again with two physiotherapists and the study team for feedback. Accordingly, the required changes were made before launching the second round. Panellists were then sent a summary of the results table including a reminder of their responses, overall group response, and a new survey questionnaire to complete approximately 8 weeks after the first round.

The second iteration was conducted from September 2021 to November 2021; the aim was to seek consensus in the areas of disagreement (≤70%) identified in the first round. Second, additional items suggested by panellists in round one were also presented for the panellists to rate. In case of disagreement, they were also asked to specify the reasoning behind their choice.

### Data analysis

Data analysis involved generating descriptive statistics (frequency and median) using a statistical package for the social sciences version 28.0. Missing data were dealt with by calculating an average score for the particular item and then replacing the missing data point with the average score.

## Results

### Response rates

The first round consisted of 30 qualified international experts from seven countries. Of these, 25 completed the second round. Therefore, the response rate for the second round was 83.3% (25 out of 30), and the attrition rate between the two rounds was 16.7% (5 out of 30).

### Panel characteristics

Table 2 summarises the panel’s demographic characteristics for each iteration. During the first round, the panellists’ average clinical and academic experience was 22.2 years (standard deviation ±12.3; range: 4–45 years) and 10.5 years (standard deviation ±10.7; range: 2–35 years), respectively.

**Table 2.** Panel's demographic characteristics.

Demographic information		Round 1 (n = 30)		Round 2 (n = 25)		Total dropouts (%)
		Frequency	%	Frequency	%	
Country of origin	UK	11	36.7	8	32	3 (10)
	USA	7	23.3	6	24	1 (3.3)
	Australia	6	20	5	20	1 (3.3
	Germany	3	10	3	12	0 (0)
	Demark	1	3.3	1	4	0 (0)
	Malaysia	1	3.3	1	4	0 (0)
	Singapore	1	3.3	1	4	0 (0)
Academic background	Physiotherapy	19	63.3	16	64	3 (10)
	Nursing	6	20	5	20	1 (3.3)
	Rehabilitation medicine	3	10	2	8	1 (3.3)
	Occupational therapy	2	6.7	2	8	0 (0)
Qualifications	Bachelors	11	36.7	8	32	3 (10)
	Masters	7	23.3	7	28	0 (0)
	FRCP	2	6.7	1	4	1 (3.3)
	PhD	8	26.7	8	32	0 (0)
	Associateship	1	3.3	1	4	0 (0)
	Diploma	1	3.3	0	0	1 (3.3)
Practice setting	Community (e.g. residential care or patient's home)	13	43.3	10	40	3 (10)
	Higher education/university	6	20	6	24	0 (0)
	Hospital (acute care)	3	10	2	8	1 (3.3)
	Hospital (long-term)	2	6.7	1	4	1 (3.3)
	Long term facility	1	3.3	1	4	0 (0)
	Rehabilitation centre	1	3.3	1	4	0 (0)
	Other	4	13.3	4	16	0 (0)

During the second round, the panellists’ average clinical and academic experience was 22.1 years (standard deviation ±11.8; range: 5–45 years) and 10.6 years (standard deviation ±10.2; range: 2–35 years), respectively.

### Development, progression, and identification of joint contractures

#### Development of joint contractures

Table 3 presents the panellists’ agreement levels regarding the importance of clinical factors associated with the development of joint contractures during each round. During the first round, 9 out of 12 items met consensus for inclusion in the core set which were: bed confinement, clonus, dystonia, impaired cognition, muscle weakness, pain, spasticity, physical function, and functional mobility. Of these 9 items, overall levels of agreement ranged between 70% and 100%. Items on which the panel consensus was below the 70% threshold in the first round were: (1) ageing, (2) pressure ulcers, and (3) urinary incontinence.

**Table 3.** Agreement levels on clinical factors.

		Round one		Round two	
	Clinical factors	Median (Average group response)	Agreement level (%)	Median (average group response)	Agreement level
Development of contractures					
1.	Ageing	5	65.5	5	80%
2.	Bed confinement	7	100	–	–
3.	Clonus	5	70	–	–
4.	Dystonia	6	86.3	–	–
5.	Impaired cognition	5	80	–	–
6.	Muscle weakness	6	100	–	–
7.	Pain	6	100	–	–
8.	Pressure ulcers	5	63.3	5	76%
9.	Spasticity	7	96.7	–	–
10.	Reduced physical function	6	96.7	–	–
11.	Reduced functional mobility	6	100	–	–
12.	Urinary incontinence	3	16.7	3	4%
13.	Postural asymmetry	–	–	5	68%
14.	Inability to engage in activities	–	–	5	76%
15.	Hypertonia	–	–	6	100%
Progression of contractures					
1.	Ageing	5	75.8	–	–
2.	Bed confinement	7	100	–	–
3.	Clonus	5	73.4	–	–
4.	Dystonia	6	86.7	–	–
5.	Impaired cognition	6	83.4	–	–
6.	Muscle weakness	6	100	–	–
7.	Pain	6	100	–	–
8.	Pressure ulcers	5	66.7	5	84%
9.	Spasticity	7	96.7	–	–
10.	Reduced physical function	6	96.7	–	–
11.	Reduced functional mobility	6	96.7	–	–
12.	Urinary incontinence	3	20.3	3	12%
13.	Postural asymmetry	–	–	5	64%
14.	Inability to engage in activities	–	–	5	76%
15.	Hypertonia	–	–	5	100%

*Suggested items*: The newly suggested items by the panellists in the first round: (1) postural asymmetry, (2) inability to engage in activities, and (3) hypertonia, were included in the second round.

In the second round, the panellists reviewed their responses in the context of the average group responses in areas of disagreement (≤70%) and rated the importance of newly suggested items. Pressure ulcers reached consensus (agreement level of 76%), while ageing and urinary incontinence still failed to reach the agreement threshold (<70%). Among the newly suggested items, inability to engage in activities and hypertonia reached an agreement level of >70% while postural asymmetry did not reach the required consensus level (68%).

#### Progression of joint contractures

Table 3 presents the panellists’ agreement levels regarding the importance of clinical factors associated with the progression of joint contractures during each round. The first round identified consensus for 10 out of 12 items. Of these, four items, including bed confinement, muscle weakness, pain, and reduced functional mobility, reached 100% agreement. On the other hand, items on which the panel consensus was below the 70% threshold were: (1) pressure ulcers and (2) urinary incontinence.

*Suggested items*: The newly suggested items by the panellists in the first round, (1) postural asymmetry, (2) inability to engage in activities, and (3) hypertonia were included in the second round.

In the second round, consensus was reached for pressure ulcers (84%), whereas urinary incontinence fell short of the agreement threshold (<70%). Among the newly proposed items, inability to engage in activities and hypertonia reached an agreement level of over 70% while postural asymmetry did not reach the required consensus level (64%).

#### Identification of joint contractures in care homes

**Figure 2.**
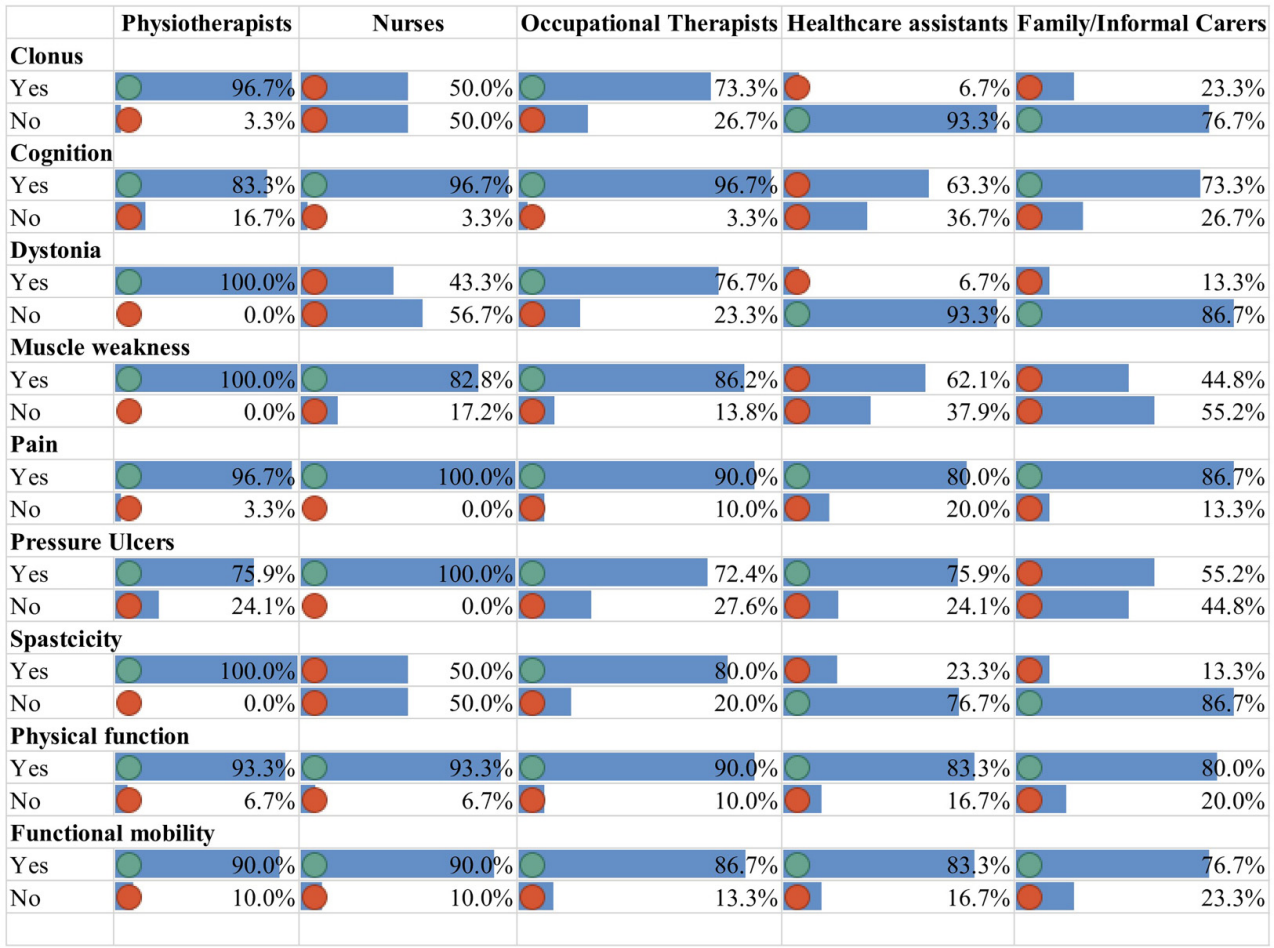
Agreement levels for the ability of healthcare professionals and carers to identify the clinical factors in care home residents.

Figure 2 shows the agreement levels of the panellists on the ability of different healthcare professionals and family/informal carers to identify the factors presented in the previous questions in adults residing in care homes. The following items failed to reach the threshold of 70% agreement for either Yes or No in round one:

- Identification of clonus, dystonia, and spasticity by nurses.
- Identification of cognitive changes and muscle weakness by care assistants.
- Identification of muscle weakness and pressure ulcers by family/informal carers.

The items which lacked consensus were sent again to the panellists to review their responses. In the second round, it was also clarified that the identification of the factors in a care home setting would be based on informal observation during the delivery of care rather than a formal clinical assessment undertaken by a specialist professional. The factors that reached consensus regarding their identification (yes) in the second round were:

- Identification of cognitive changes and muscle weakness by care assistants.
- Identification of muscle weakness, and pressure ulcers by family/informal carers.

Of the new factors suggested by the panellists namely, postural asymmetry, activity engagement, and hypertonia reached an agreement for all healthcare professionals except the identification of postural asymmetry by healthcare assistants and family/informal carers.

#### Contextual factors

Table 4 presents the expert panel’s agreement levels regarding the contextual factors relevant to the development of joint contractures during each round. During the first round, there was a group consensus for 8 out of 9 items. Of these 8 items, overall levels of agreement ranged between 86.6% and 100%. Diet was the only item on which the panel consensus was below the 70% threshold in the first round. Panellists were also asked to specify any other contextual factors that were missing from the list in their opinion. The newly suggested items were (1) education and training of caregivers, (2) staffing levels in the care home, (3) service user training and involvement, and (4) timely access to and quality of in-reaching services. In round two, diet still did not reach the agreement threshold (<70%), while all newly suggested items gained consensus (92%).

**Table 4.** Agreement level on contextual factors.

		Round one		Round two	
	Contextual factors	Median (average group response)	Agreement level (%)	Median (average group response)	Agreement level
1.	Diet	4.5	50	4	24%
2.	Inappropriate design of assistive devices	5	89.6	–	–
3.	Lack of regular social engagement (friends, family, community members)	5	70	–	–
4.	Lack of support from immediate family	5	86.6	–	–
5.	Lack of support from healthcare professionals (physiotherapists, nurses, occupational therapists)	7	96.7	–	–
6.	Lack of support from healthcare assistants	6	93.3	–	–
7.	Medication	6	93.1	–	–
8.	Organisation and policies of the care home	6	90	5	–
9.	Resources of the care home	7	100	–	–
10.	Education and training of caregivers	–	–	6	92%
11.	Staffing levels	–	–	6	92%
12.	Service user training and involvement	–	–	6	92%
13.	Timely access to and quality of in-reaching services	–	–	6	92%

#### Preventive care approaches

Table 5 presents the expert panel's agreement levels regarding the care approaches important in the prevention of the development and progression of joint contractures during each round. During the first round, there was a consensus for 7 out of 8 care approaches. Of these seven approaches, overall levels of agreement ranged between 70% and 100%. The only care approach on which the panel consensus was below the 75% threshold in the first round was ‘passive exercises’.

**Table 5.** Agreement levels on preventive care approaches.

		Round one		Round two	
	Preventive care approaches	Median (average group response)	Agreement level (%)	Median (average group response)	Agreement level
1.	Encouraging to sit, transfer, move around, exercise, and perform activities of daily living with minimal possible assistance	7	100	–	–
2.	Ensuring adequate nutrition and hydration.	6	73.3	–	–
3.	Identifying and managing skin irritations and rashes	6	70	–	–
4.	Performing passive exercises	6	66.7	5	60%
5.	Performing stretching exercises	6	76.7	–	–
6.	Postural management /positioning techniques	7	90	–	–
7.	Taking appropriate action to reduce or minimise pain or discomfort.	7	100	–	–
8.	Using appropriate products to prevent or manage skin breakdown	7	90	–	–
9.	24-h postural management program	–	–	6	76%
10.	Caregiver/service user education and training	–	–	6	84%

Expert panellists were also asked to specify any other preventive care approaches that were missing from the list. The most common new approaches identified were a 24-hour postural management program and caregiver/service user education and training.

In the second round, passive exercises remained below the agreement threshold (<70%), while both newly suggested items gained consensus (76% and 84%, respectively).

## Discussion

This e-Delphi survey generated expert opinions and consensus on items providing a provisional framework for the development of a contracture risk assessment tool (ORACLE) for adults in care homes.

This Delphi survey sought expert opinion and consensus on three aspects of the ORACLE: (1) *Clinical factors* that form the first part of ORACLE, (2) *Preventive care approaches* that form the second part of the ORACLE, and (3) *Contextual factors* that will be used to develop a guidance manual for ORACLE to improve its usability and practical implementation in a care home setting. Table 6 shows a list of finally agreed items for ORACLE by the study team.

**Table 6.** Finally agreed items for Observational Risk Assessment Tool for Contractures: Longitudinal Evaluation (ORACLE).

Clinical factors	Contextual factors	Preventive care approaches
Ageing Bed confinement Impaired cognition Muscle weakness Pain Pressure ulcers Reduced physical function Reduced functional mobility Inability to engage in activities	Inappropriate design of assistive devices Lack of regular social engagement Lack of support from immediate family Lack of support from healthcare professionals Lack of support from healthcare assistants Medication Organisation and policies of the care home resources of the care home Education and training of caregivers Staffing levels Service user training and involvement Timely access to and quality of in-reaching services	Encouraging to sit, transfer, move around, exercise, and perform activities of daily living with minimal possible assistance Ensuring adequate nutrition and hydration Identifying and managing skin irritations and rashes Performing stretching exercises Postural management /positioning techniques Taking appropriate action to reduce or minimise pain or discomfort. Using appropriate products to prevent or manage skin breakdown 24-h postural program

The category, clinical factors encompassed factors associated with the development and progression of joint contractures. Of the total 15 items, ≥70% of panellists provided agreement on 10 items of which spasticity is one.

However, there was insufficient panel consensus regarding spasticity being recognisable by the care assistants, the aimed primary users of the tool in the care home. Spasticity is a commonly reported secondary complication following chronic neurological conditions (e.g. stroke) in care home residents.^31^ Identifying changes in muscle tone, including spasticity, requires physical and neurological examination by a trained practitioner; the primary care providers at the care home facilities might lack the appropriate training to recognise it^32^; therefore, it was excluded from the final list of items for ORACLE. Interestingly, our systematic review also reported that the evidence on the relationship between spasticity and contractures remains unclear and inconclusive.^21^

The category, preventive care approaches included approaches important in preventing contracture development and progression. This forms the second part of ORACLE, which provides guidance to the care home staff to prescribe a set of actions in response to the level of risk identified in the first part. The panel demonstrated a high level of consensus for 9 out of 10 care approaches. The only care approach which failed to reach adequate panel consensus was ‘performing passive exercises’. Passive exercises are a common intervention for individuals at risk of developing contractures.^33^ When asked by the panellists about the reasoning behind their choice, a few stated that there is insufficient evidence to support its effectiveness in preventing contractures. The panel views are consistent with the findings of a systematic Cochrane review which provides inconclusive evidence of passive movements as an effective treatment approach to prevent or manage contractures.^33^ Moreover, a recent systematic review that investigated nonsurgical treatment options for muscle contractures in neurological disorders could not provide convincing evidence for using passive movements.^34^ Therefore, ‘passive exercises’ were excluded from the final list of items. Contrastingly, panellists provided consensus in favour of stretching; however, recent systematic reviews have substantiated that stretching did not produce clinically important short-term effects on joint mobility.^34,35^ Further research is needed to investigate the long-term effects of stretching on joint mobility and the prevention of contractures. Given this contradiction, the study team suggested that it was not appropriate to recommend that non-physiotherapeutic staff engages in the application of stretching exercises on their own; therefore, it was also excluded.

While contextual factors are not included in the tool, they are relevant to its successful implementation. The internal quality of an intervention is only one factor in its effectiveness, which will also depend upon the intervention's interaction with the environment in which it has been introduced and upon the responses of the actors involved.^36^ This is especially the case in complex environments such as care homes.^37^ A growing body of evidence supports the need for context-specific research studies to successfully implement complex interventions.^38,39^ A recent systematic review by Peryer et al.^37^ has reported that several large-scale studies evaluating complex interventions in care homes have demonstrated inconclusive or neutral findings. It is unclear whether the findings are linked solely to the ineffective interventions or contextual barriers around their implementation.^37^ The contextual mechanisms identified by the panellists in this study will be included in the ORACLE guidance manual with a view to mitigating their inhibitive effects on the effectiveness of the tool. Panellists provided agreement on 12 out of 13 contextual factors.

The current study has several key strengths. It included panellists from diverse geographical locations, cultural settings, and academic backgrounds, with 50% having at least a higher degree and 50% having at least one research publication on joint contractures, indicating a broad range of representation of skills and diversity of expertise. The number of rounds, consensus method, and the level of agreement were defined a priori which is in line with recommendations for conducting and reporting of Delphi studies.^20^ Two iterations ensured that the panellists could revisit their opinions on the pre-determined list of items, suggest additional items that were missing in their opinion, and reach an agreement. Another strength of the current study was that the survey questionnaire was designed using a pre-determined list of items derived from a range of sources, including peer-reviewed research evidence, which may have reduced the researcher bias. Moreover, this study piloted the survey questionnaire prior to the launch of the study to ensure good face and content validity.

One of the limitations of this study was that the selection of the panel experts was restricted to those who could understand and write English. As a result, potential non-English panel experts were excluded.

Building on the extant research literature, this Delphi study determined expert consensus on items to be included in a contracture risk assessment tool (ORACLE). The next step is to evaluate the reliability, acceptability, and usability of the tool within care homes. A valid and reliable contracture risk assessment tool might have the potential to trigger timely and appropriate referrals. Timely referrals may aid in prompt escalation of early interventions by the specialists aiming to reduce the risk of contracture development or progression of existing contractures.

**Clinical messages**

- An important strategy to prevent joint contractures is to systematically identify the risk of their development.
- The expert panel identified the key clinical factors that contribute to this risk and provided strategies to prevent their development and progression.
- The expert panel highly recommends the preservation of mobility and functional independence as a key priority for reducing risk of contractures.
- Notwithstanding the strength of the evidence generated by this survey, an effective risk assessment tool is required to ensure a systematic approach to early intervention and prevention of joint contractures.
